# Fatty metaplasia quantification and impact on regional myocardial function as assessed by advanced cardiac MR imaging

**DOI:** 10.1007/s10334-017-0639-7

**Published:** 2017-06-15

**Authors:** Tomas Lapinskas, Bernhard Schnackenburg, Marc Kouwenhoven, Rolf Gebker, Alexander Berger, Remigijus Zaliunas, Burkert Pieske, Sebastian Kelle

**Affiliations:** 1 Department of Cardiology, Medical Academy, Lithuanian University of Health Sciences, Eiveniu Street 2, 50161 Kaunas, Lithuania; 2 Department of Internal Medicine/Cardiology, German Heart Institute Berlin, Augustenburger Platz 1, 13353 Berlin, Germany; 3 Philips Healthcare, Röntgenstraße 24, 22335 Hamburg, Germany; 4 Philips Healthcare, Veenpluis 4-6, 5684 PC Best, The Netherlands; 5 DZHK (German Centre for Cardiovascular Research), Partner Site, Berlin, Germany

**Keywords:** Cardiac magnetic resonance, Chronic myocardial infarction, Fatty metaplasia, Feature tracking, Fat–water separated imaging

## Abstract

**Objective:**

This study aimed to investigate the advantages of recently developed cardiac imaging techniques of fat–water separation and feature tracking to characterize better individuals with chronic myocardial infarction (MI).

**Materials and methods:**

Twenty patients who had a previous MI underwent CMR imaging. The study protocol included routine cine and late gadolinium enhancement (LGE) technique. In addition, mDixon LGE imaging was performed in every patient. Left ventricular (LV) circumferential (Ecc_LV_) and radial (Err_LV_) strain were calculated using dedicated software (CMR^42^, Circle, Calgary, Canada). The extent of global scar was measured in LGE and fat–water separated images to compare conventional and recent CMR imaging techniques.

**Results:**

The infarct size derived from conventional LGE and fat–water separated images was similar. However, detection of lipomatous metaplasia was only possible with mDixon imaging. Subjects with fat deposition demonstrated a significantly smaller percentage of fibrosis than those without fat (10.68 ± 5.07% vs. 13.83 ± 6.30%; *p* = 0.005). There was no significant difference in Ecc_LV_ or Err_LV_ between myocardial segments containing fibrosis only and fibrosis with fat. However, Ecc_LV_ and Err_LV_ values were significantly higher in myocardial segments adjacent to fibrosis with fat deposition than in those adjacent to LGE only.

**Conclusions:**

Advanced CMR imaging ensures more detailed tissue characterization in patients with chronic MI without a relevant increase in imaging and post-processing time. Fatty metaplasia may influence regional myocardial deformation especially in the myocardial segments adjacent to scar tissue. A simplified and shortened myocardial viability CMR protocol might be useful to better characterize and stratify patients with chronic MI.

## Introduction

The prognosis of subjects who survived acute myocardial infarction (MI) has substantially improved in recent decades [1]. Myocardial healing after acute damage is an active process and changes in myocardial tissue composition may influence cardiac function as well as future events and mortality [2].

A recent observational cardiac magnetic resonance (CMR) study reported that fat deposition can be detected in up to 78% of individuals who experienced MI [3]. Lipomatous metaplasia, also termed fat infiltration, is associated with more adverse cardiac remodeling and larger infarct size [4]. Moreover, it has been noted recently that these structural changes in the extracellular matrix of the myocardium increase the risk of ventricular arrhythmias and sudden cardiac death. Therefore, noninvasive detection of this myocardial remodeling could have great prognostic value [5].

Validated CMR technique using a three-dimensional (3D) single breath-hold ECG-gated magnetization prepared multiecho Dixon (mDixon) sequence has gained increased interest over recent years. Fat-only images derived using this technique can be used to detect and quantify cardiac adipose tissue [6]. The proposed fat–water separation (mDixon) method has a number of benefits, such as excellent discrimination between fat and water, improved diagnostic confidence and better signal-to-noise ratio (SNR). It, therefore, shows the potential to replace conventional fat imaging techniques, while the simultaneously acquired water-only images may be a replacement for conventional imaging, including late gadolinium enhancement (LGE) [7–9].

The CMR feature tracking (CMR-FT) technique provides information about myocardial mechanics. Myocardial strain and strain rate can be derived from conventional balanced steady state free precession (bSSFP) cine images and can be used to assess myocardial function [10]. Recent studies report excellent inter-observer and intra-observer agreement and high inter-study reproducibility of the technique to quantify myocardial deformation [11–13].

We conducted this study to determine the advantages of recent CMR imaging techniques. We assessed changes in myocardial tissue after MI using mDixon technique. In addition, we investigated changes in global and regional myocardial deformation in patients with lipomatous metaplasia using CMR-FT.

## Materials and methods

### Study population

We retrospectively enrolled 20 subjects with chronic MI (infarct age median 60.0 months; range 13.0–90.0 months) to assess global and regional cardiac function, myocardial tissue composition, viability, and new myocardial ischemia. The medical history of the participants was obtained from medical records. The study complies with the Declaration of Helsinki and was performed in accordance with local law. Informed consent was obtained from all patients.

### Cardiac magnetic resonance

All CMR images were acquired using 1.5 (Achieva) or 3 T (Ingenia, Philips Healthcare, Best, the Netherlands) MRI scanners with a 32-channel cardiac surface coil in supine position. All study participants were scanned using an identical comprehensive imaging protocol.

The study protocol included initial scouts to determine cardiac imaging planes. Cine images were acquired using ECG-gated bSSFP sequence with multiple breath-holds at end-expiration in three left ventricular (LV) long-axis (two-chamber, three-chamber, and four-chamber) planes. The ventricular two-chamber and four-chamber planes were used to plan the stack of short-axis slices covering the entire LV. The following imaging parameters were used. For the 1.5 T scanner: repetition time (TR) = 3.3 ms, echo time (TE) = 1.6 ms, flip angle = 60°, acquisition voxel size = 1.8 × 1.7 × 8.0 mm^3^ , and 30 phases per cardiac cycle; for the 3 T scanner: TR = 2.9 ms, TE = 1.45 ms, flip angle = 45°, acquisition voxel size = 1.9 × 1.9 × 8.0 mm^3^ , and 30 phases per cardiac cycle.

**Fig. 1:**
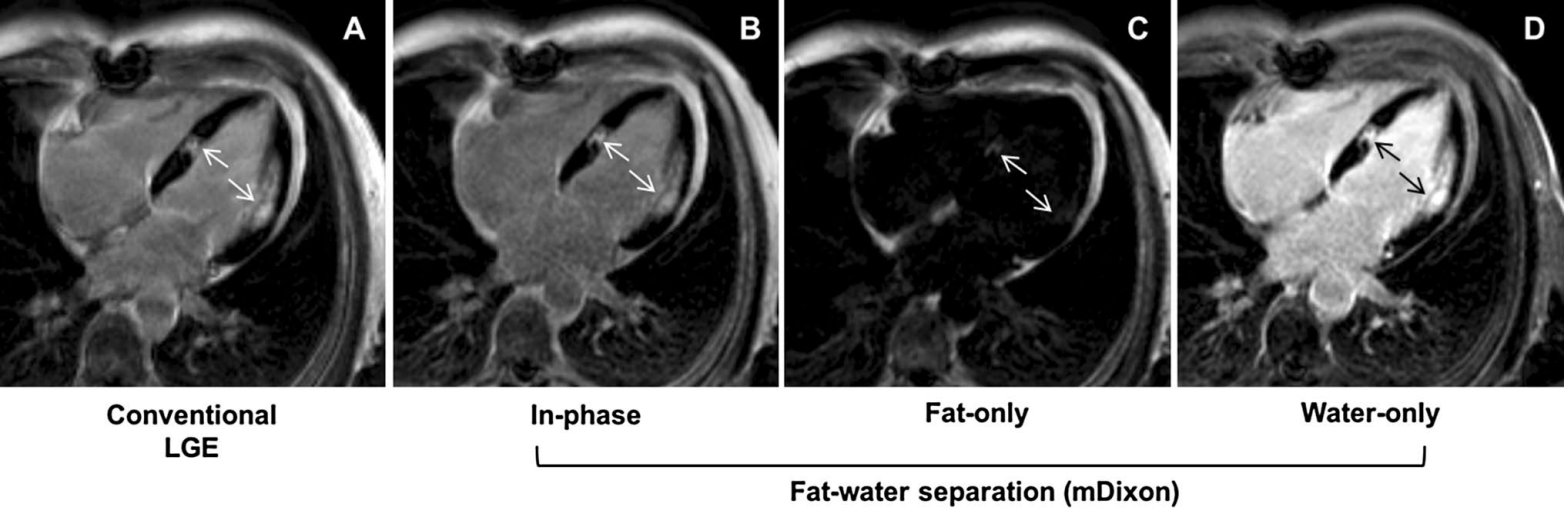
Example of patient with history of two chronic MI. Conventional LGE (**a**), in-phase (**b**), fat-only (**c**), and water-only (**d**) mDixon images acquired in ventricular four-chamber view. LGE, in-phase and water-only mDixon images demonstrate scar tissue in the medial inferoseptal and medial anterolateral segments (*arrows*). Fat deposition is visible in fat-only image in corresponding myocardial segments (*arrows*). *MI* myocardial infarction, *LGE* late gadolinium enhancement, *mDixon* multiecho inversion recovery spoiled gradient echo

The LGE images were obtained 10 min after the injection of 0.15 mmol/kg gadobutrol (Gadovist^®^, Bayer Schering Pharma AG, Berlin, Germany). A Look-Locker sequence was acquired to determine the inversion time to null the signal of the LV myocardium. A 3D inversion recovery fat saturated spoiled gradient echo sequence was used to detect scar tissue in three LV long-axis and short-axis orientations (Fig. 1a). Typical parameters for imaging were the following. For the 1.5 T scanner: TR = 3.3 ms, TE = 1.6 ms, flip angle 15°, acquisition voxel size 1.6 × 1.6 × 10.0 mm^3^, parallel imaging factor (SENSE) = 2.2; for the 3 T scanner: TR = 3.2 ms, TE = 1.62 ms, flip angle 15°, acquisition voxel size 1.5 × 1.5 × 10.0 mm^3^, SENSE = 2.2. The breath-hold time varied from 12 to 16 s, depending on anthropometrics. Single 3D long-axis scan was acquired during one breath-hold. Two breath-holds were required to acquire full 3D short-axis scan.

Additionally, in all study participants a single breath-hold ECG-gated 3D inversion recovery spoiled gradient multiecho (mDixon) sequence was used for fat water separation imaging. The following sequence parameters were used. For the 1.5 T scanner: TR = 4.7 ms, TE1 = 1.5 ms, TE2 = 3.0 ms, flip angle = 15°, voxel size 1.6 × 1.6 × 5.0 mm^3^, parallel imaging factor (SENSE) = 2.2; for the 3 T scanner: TR = 3.8 ms, TE1 = 1.3 ms, TE2 = 2.4 ms, flip angle = 12°, voxel size 1.7 × 1.7 × 5.0 mm^3^, SENSE = 2.2. In-phase (Fig. 1b), fat-only (Fig. 1c), and water-only (Fig. 1d) images were reconstructed at the scanner.

### Image analysis

All images were analyzed offline using commercially available software (Medis Suite, version 2.0, Leiden, the Netherlands) in accordance to a recent consensus document for quantification of LV function and mass using CMR [14]. LV end-diastolic (LV EDV) and end-systolic (LV ESV) volumes were quantified using manual planimetry of the endocardial and epicardial surface from short-axis stack and LV ejection fraction (LV EF); myocardial mass, cardiac output and cardiac index were calculated. Papillary muscles were considered part of the blood pool. LV volumes and myocardial mass were adjusted to body surface area which was calculated by the Mosteller method.

The endocardial and epicardial contours drawn on cine images were transferred into LGE images. The presence and extent of LGE were quantified using the signal threshold versus reference mean (STRM) >3 standard deviations (SD) method as it provides the greatest accuracy with acceptable reproducibility compared with other signal intensity threshold techniques [15]. All algorithm-selected pixels in the myocardium were counted on each of the LGE images. The total LGE volume and mass were calculated automatically. The extent of scar tissue was defined using a 5-point scale where 0 = absence of LGE; 1 = LGE of 1–25% of LV wall thickness; 2 = LGE extending to 26–50%; 3 = LGE extending to 51–75%; and 4 = LGE extending to 76–100% [16].

The endocardial and epicardial contours were similarly transferred into mDixon images using the same program. The fat deposition volume and mass were calculated using the same signal intensity threshold level (>3 SD) in the fat-only images. The extent of fat was classified as subendocardial, subepicardial and transmural. Fibrosis volume and mass were calculated using the identical approach in the water-only images. Additionally, the extent of LGE was assessed in the in-phase mDixon images. The location of LGE and fat deposition was defined using a standard American Heart Association (AHA) 17-segment model [17]. The global LGE (fibrosis plus fat) and fibrosis as percentages of LV mass and the fatty metaplasia as percentage of LV mass and global LGE mass were calculated.

The cine images were used to calculate myocardial circumferential (Ecc_LV_) and radial (Err_LV_) strain using commercially available software CMR^42^ (Circle Cardiovascular Imaging Inc., Calgary, Canada). LV endocardial and epicardial borders were contoured by a point-and-click approach in three short-axis slices (basal, mid-ventricular, and apical) at LV end-diastolic phase. After application of a tissue tracking algorithm endocardial and epicardial borders were detected through all cardiac phases. The right ventricular upper septal insertion point was manually defined to allow accurate segmentation according to an AHA 16-segment model. LV strain analysis was performed on a segmental level. Segments with LGE extent of <50% of LV wall thickness were excluded from analysis, because the sensitivity and specificity of the method to detect myocardial segments with scar is highest when LGE extent is >50% of LV wall thickness [18]. All image analysis was performed by two experienced (CMR level 3) investigators.

### Statistical analysis

Data analysis was performed using Microsoft Excel and IBM SPSS Statistics version 23.0 software (SPSS Inc., Chicago, IL, USA) for Windows. The Shapiro–Wilk test was used to determine whether the data were normally distributed. Continuous variables were expressed as mean ± SD or median [interquartile range] depending on their distribution, and categorical variables were described as total number (percentage). Differences in normally and non-normally distributed continuous variables were established using an unpaired Student *t* test and Mann–Whitney *U* test, respectively. A *p* value <0.05 was considered to indicate statistically significant difference.

## Results

### Study population

**Fig. 2:**
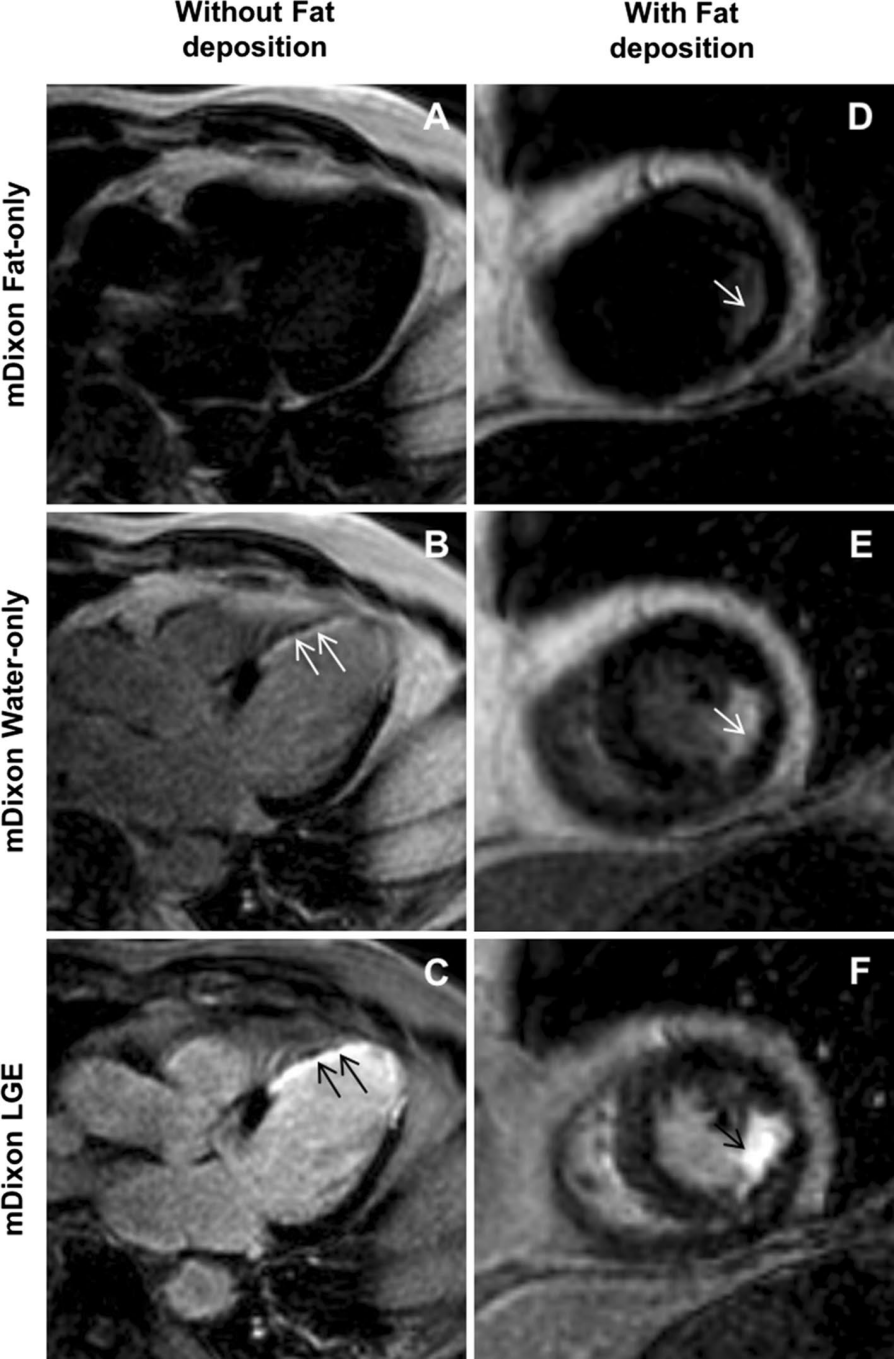
The* left column* three images are from a patient with chronic transmural MI without fat deposition: fat-only (**a**), water-only (**b**), and in-phase (**c**) mDixon images acquired in ventricular three-chamber view. Water-only and in-phase mDixon images demonstrate transmural scar (*arrows*) in the medial anteroseptal, apical septal and lateral segments as well as apical cap. However, despite transmural extent of scar tissue fat-only mDixon image did not show fat accumulation. The *right column* three images in short-axis orientation are from patient with subendocardial scar and fat deposition: Fat-only (**d**) mDixon image shows fatty metaplasia in the medial inferolateral segment. Scar tissue is nicely depicted in water-only (**e**) and in-phase (**f**) mDixon images. *MI* myocardial infarction; *mDixon* multiecho inversion recovery spoiled gradient echo

In total, 20 patients (10 with fat deposition and 10 without) were included in our study. All participants were scanned using two different magnetic field strength MRI scanners (1.5 and 3 T) at random without any specific selection. All patients without fat deposition were imaged by the 1.5 T machine, whereas three (30%) patients with fat deposition underwent CMR imaging on the 3 T scanner. There was no significant difference between the groups with respect to subject age, gender, body mass index, body surface area, or infarct age. Figure 2 represents two patients with chronic MI, but only one demonstrates fatty metaplasia (2D, 2E, and 2F). Table 1 summarizes the demographic and functional characteristics of the study population.

**Table 1:** Subject characteristics

	Fat deposition absent group (*n* = 10)	Fat deposition present group (*n* = 10)	*p* value
Demographics			
Age (years)	64.40 ± 9.34	58.00 ± 10.41	0.165
Male gender	9 (90%)	9 (90%)	1.000
BMI (kg/m^2^)	27.84 ± 2.81	28.16 ± 3.37	0.822
BSA (m^2^)	2.02 ± 0.18	2.09 ± 0.16	0.387
Infarct age (months)	15.0 [8.5–91.0]	72.0 [51.0–145.5]	0.143
Volumetric and functional parameters			
LV EDV (mL)	175.75 ± 33.42	166.70 ± 37.92	0.578
LV EDV index (ml/m^2^)	86.85 ± 14.39	80.78 ± 23.12	0.492
LV ESV (mL)	80.40 ± 24.64	85.10 ± 29.32	0.703
LV ESV index (ml/m^2^)	39.74 ± 11.83	41.39 ± 16.87	0.803
LV EF (%)	55.07 ± 8.90	49.86 ± 7.20	0.168
LV mass (g)	113.14 ± 21.51	105.70 ± 16.27	0.396
LV mass index (g/m^2^)	55.77 ± 8.54	50.56 ± 6.62	0.146
Cardiac output (L/min)	6.57 ± 1.23	5.71 ± 1.06	0.112
Cardiac index (L/min/m^2^)	3.24 ± 0.51	2.73 ± 0.44	0.029

Results are reported as mean ± standard deviation, total number (percentage), or median [interquartile rage]

*BMI* body mass index, *BSA* body surface area, *LV* left ventricle/ventricular, *EDV* end-diastolic volume, *ESV* end-systolic volume, *EF* ejection fraction

### Global cardiac function

In our study, LV EDV, LV EDV index, LV ESV, LV ESV index, LV mass, LV mass index, and cardiac output were similar in both groups. A trend of lower LV EF was seen in patients with detected lipid accumulation, but the difference did not reach statistical significance (55.07 ± 8.90% vs. 49.86 ± 7.20%; *p* = 0.168). Subjects with fat deposition had significantly lower cardiac index compared with those without fatty metaplasia (2.73 ± 0.44 L/min/m^2^ vs. 3.24 ± 0.51 L/min/m^2^; *p* = 0.029).

### Scar and fatty metaplasia analysis

Scar and fat deposition were analyzed using a standard AHA 17-segment model (17 segments in 20 patients resulting in 340 segments).

Among the total of 340 segments analyzed in this study, 91 (26.8%) had LGE and 31 (9.1%) had fat deposition. Twenty-seven (7.9%) segments had both LGE and fat deposition, while 64 (18.8%) segments had LGE and no fat deposition. Of 170 segments analyzed in the fat deposition group, four (1.2%) segments had fat deposition without LGE, but all these segments were adjacent to myocardial segments with LGE.

Of 20 infarcts, 18 (90%) were transmural and the remaining two (10%) were subendocardial. All patients with fat deposition had transmural myocardial infarctions. Of 10 transmural infarcts in the fat deposition group, two (20%) had fat deposition in the subendocardial layer and two (20%) in the subepicardial layer of myocardium. The remaining six (60%) patients showed transmural fat deposition.

Patients with fatty metaplasia had significantly more segments with LGE than patients without lipomatous metaplasia (5.50 ± 2.46 segments vs. 3.60 ± 1.26 segments; *p* = 0.048). Significantly higher LGE mass was found in patients with fat deposition (19.12 ± 7.48 g vs. 11.23 ± 6.01 g; *p* = 0.019), as well as a larger percentage of LGE (17.77 ± 5.49% vs. 9.88 ± 4.37%, *p* = 0.004). The fibrosis mass and percentage to myocardial mass were similar in both groups (Table 2).

**Table 2:** Comparison of myocardial tissue characterization between study subjects

	Fat deposition absent group (*n* = 10)	Fat deposition present group (*n* = 10)	*p* value
Global LGE (fibrosis + fat)			
Mass (g)	11.23 ± 6.01	19.12 ± 7.48	0.019
Ratio of LVM (%)	9.88 ± 4.37	17.77 ± 5.49	0.004
Fibrosis			
Mass (g)	11.23 ± 6.01	12.46 ± 6.99	0.796
Ratio of LVM (%)	9.88 ± 4.37	11.49 ± 5.82	0.529
Fat deposition			
Mass (g)	0.00	5.17 ± 2.61	–
Ratio of LVM (%)	0.00	4.83 ± 2.33	–
Ratio of LGE mass (%)	0.00	27.30 ± 9.96	–

Results are expressed as mean ± standard deviation

*LGE* late gadolinium enhancement, *LVM* left ventricular mass

**Fig. 3:**
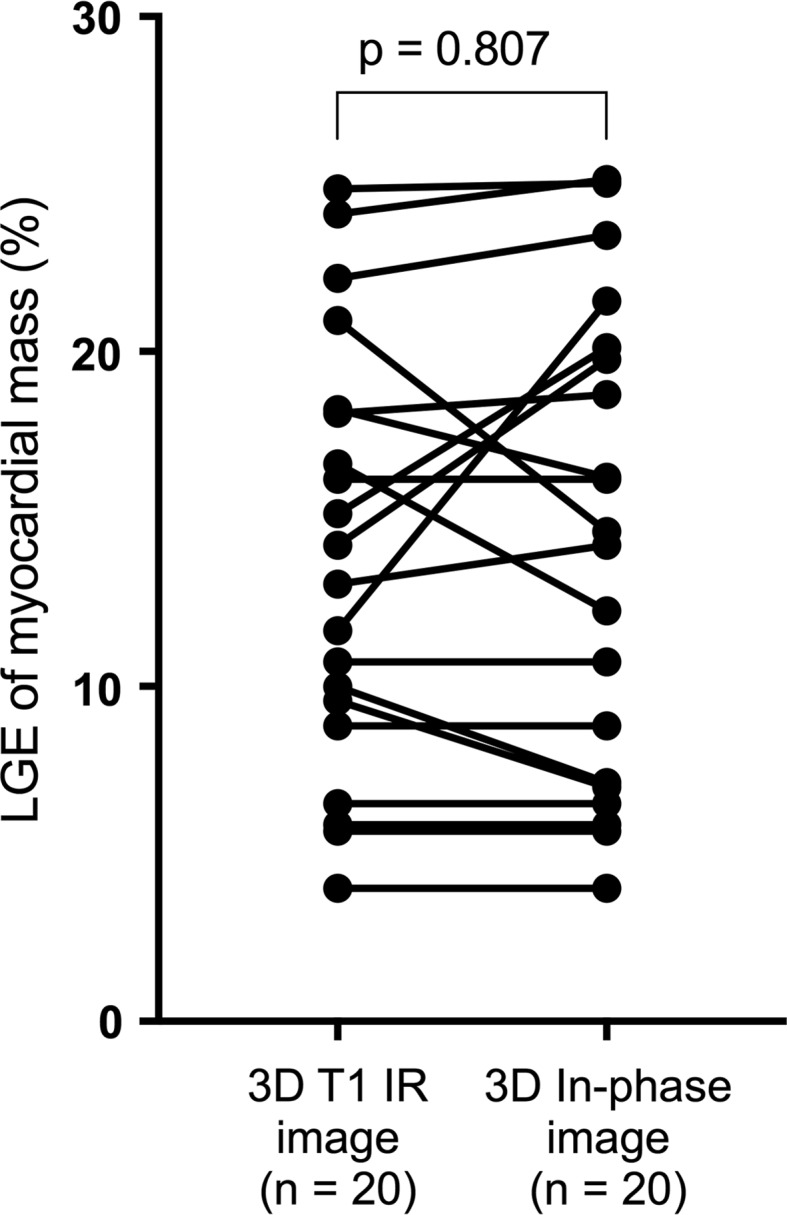
The infarct size estimated in conventional LGE images is similar to that calculated in mDixon images. *LGE* late gadolinium enhancement, *mDixon* multiecho inversion recovery spoiled gradient echo

The infarct size measured in conventional LGE images was similar when compared with those estimated in in-phase (mDixon) images (15.17 ± 7.75 g vs. 15.58 ± 8.59 g; *p* = 0.807). The percentage of infarcted myocardium was also similar for both imaging techniques (*p* = 0.807) (Fig. 3). Individuals with fat deposition had significantly smaller percentage of fibrosis than those without (10.68 ± 5.07% vs. 13.83 ± 6.30%; *p* = 0.005). Detection of fat deposition was only possible with fat–water separated (mDixon) imaging. Fat deposition mass and percentage to LV myocardial mass in patients with lipomatous metaplasia were 5.17 ± 2.61 g and 4.83 ± 2.33%, respectively (Table 3).

**Table 3:** Comparison of myocardial tissue characteristics parameters between two methods

	3D T1 Inversion recovery (*n* = 20)	3D In-phase (mDixon) (*n* = 20)	*p* value
Global LGE			
Mass (g)	15.17 ± 7.75	15.58 ± 8.59	0.807
Ratio of LVM (%)	13.83 ± 6.30	14.15 ± 6.97	0.807
Fibrosis			
Mass (g)	15.17 ± 7.75	12.99 ± 7.68	0.101
Ratio of LVM (%)	13.83 ± 6.30	10.68 ± 5.07	0.005
Fat deposition			
Mass (g)	0.00	5.17 ± 2.61	–
Ratio of LVM (%)	0.00	4.83 ± 2.33	–

Results are expressed as mean ± standard deviation. Fat deposition mass and fat deposition as percentage of myocardial mass were calculated only in patients with fat deposition (*n* = 10)

*3D* three dimensional, *LGE* late gadolinium enhancement, *LVM* left ventricular mass

Seven (70%) patients with fat deposition detected using mDixon imaging had dark zones in the myocardium in bSSFP cine images due to fat tissue induced chemical-shift artifacts.

### Myocardial deformation analysis

As described in the methods, analysis of segmental myocardial deformation was performed using an AHA 16-segment model (16 segments in 20 patients resulting in 320 segments).

Among a total of 320 segments analyzed, 85 (26.6%) had LGE and 31 (9.7%) had fat deposition. Twenty-seven (8.4%) segments had both LGE and fat deposition, while 58 (18.1%) had LGE and no lipomatous metaplasia. Four (1.3%) segments had fat deposition without LGE.

**Fig. 4:**
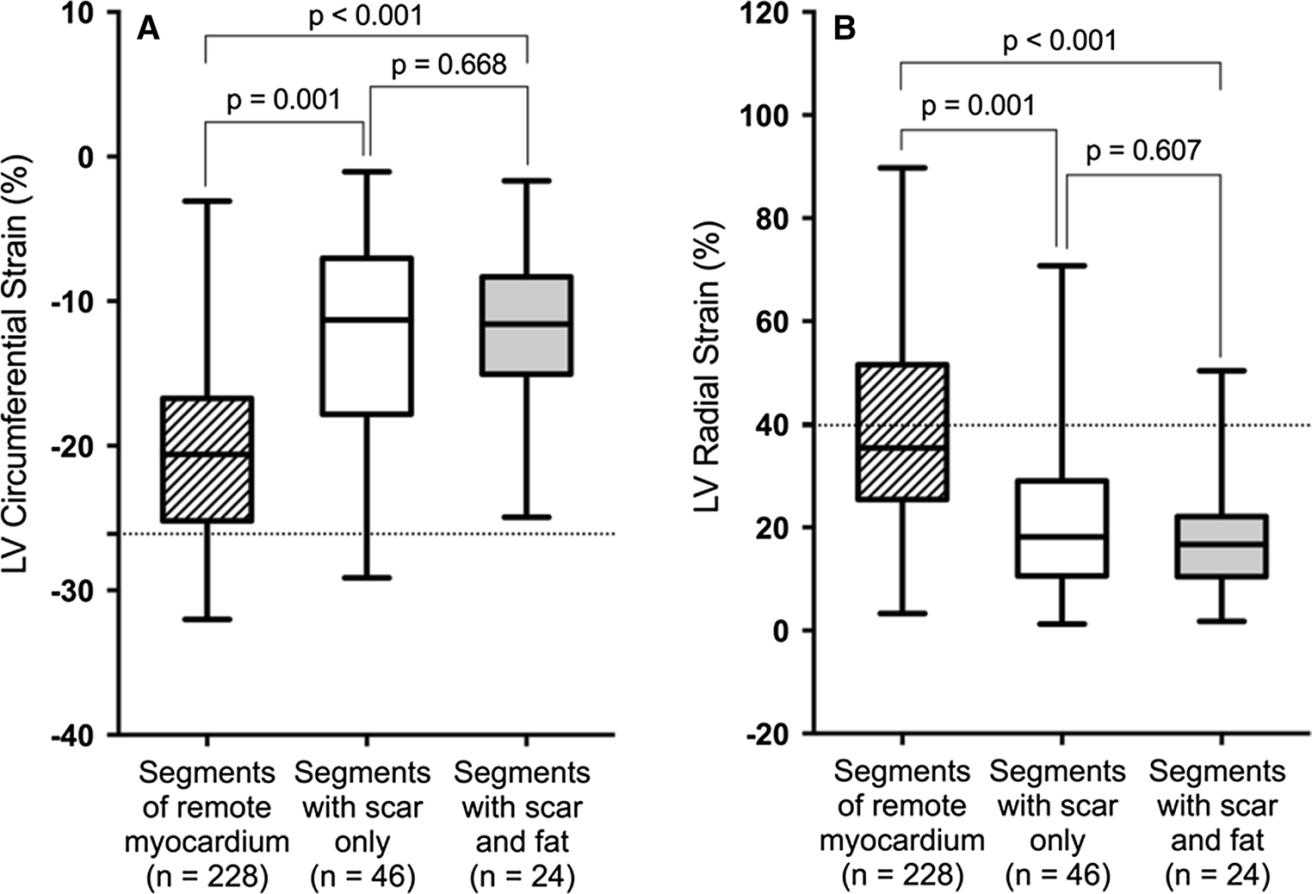
Comparison of LV circumferential (**a**) and radial (**b**) strain between segments of remote myocardium and segments containing fibrosis only or fibrosis and fat tissue. The Ecc_LV_ and Err_LV_ values were significantly higher in segments of remote myocardium than in segments containing fibrosis only or fibrosis and fat. There was no significant difference in strain parameters between segments containing fibrosis only and fibrosis and fat. *LV* left ventricular, *Ecc*_*LV*_ left ventricular circumferential strain, *Err*_*LV*_ left ventricular radial strain. *Dotted lines* correspond normal values of myocardial strain measurements derived using CMR feature tracking [36]

**Fig. 5:**
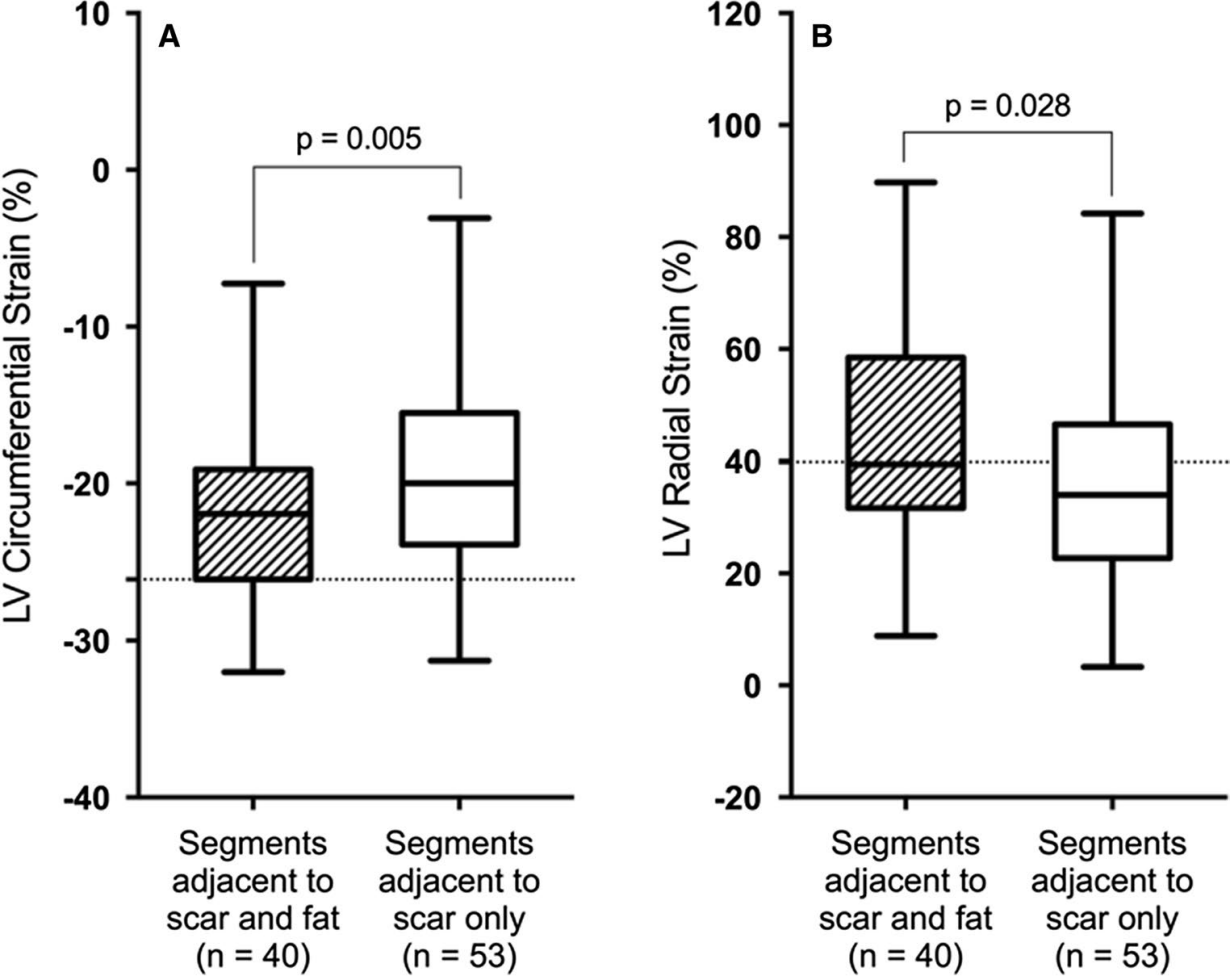
Comparison of LV circumferential (**a**) and radial (**b**) strain values between myocardial segments adjacent to fibrosis and fat deposition and segments adjacent to fibrosis only. The Ecc_LV_ and Err_LV_ strain values were significantly higher in myocardial segments adjacent to segments containing fibrosis and fat tissue. *LV* left ventricular, *Ecc*_*LV*_ left ventricular circumferential strain, *Err*_*LV*_ left ventricular radial strain. *Dotted lines* correspond normal values of myocardial strain measurements derived using CMR feature tracking [36]

There were significantly lower Ecc_LV_ and Err_LV_ values of segments with LGE when compared with segments of remote myocardium (−12.39 ± 6.71% vs. −20.37 ± 6.11%; *p* < 0.001 for Ecc_LV_ and 19.84 ± 15.19% vs. 39.11 ± 18.63%; *p* < 0.001 for Err_LV_, respectively). There was no significant difference in Ecc_LV_ or Err_LV_ between segments containing fibrosis only and fibrosis with fat deposition (−11.94 ± 5.92% vs. −12.63 ± 7.14%; *p* = 0.668 for Ecc_LV_ and 20.85 ± 16.48% vs. 17.89 ± 12.43%; *p* = 0.607 for Err_LV_, respectively) (Fig. 4a, b). The global Ecc_LV_ and Err_LV_ values were similar in both groups (−16.30 ± 1.55% vs. −16.94 ± 3.11%; *p* = 0.799 for global Ecc_LV_ and 29.43 ± 2.85% vs. 29.97 ± 8.82%; *p* = 0.959 for global Err_LV_, respectively). Interestingly, there were significantly higher Ecc_LV_ and Err_LV_ values of myocardial segments adjacent to segments containing fibrosis and fat deposition than in those adjacent to segments containing fibrosis only (−22.27 ± 4.97% vs. −19.40 ± 6.87%; *p* = 0.005 for Ecc_LV_ and 44.55 ± 17.42% vs. 36.90 ± 19.92%; *p* = 0.028 for Err_LV_, respectively) (Fig. 5a, b).

## Discussion

In this single center study we noninvasively assessed myocardial tissue characteristics in patients with old MI using conventional and newly developed CMR imaging—mDixon technique. We assessed the relationship between fatty metaplasia and global as well as regional cardiac function parameters. Our study is based on intra-individual and inter-individual comparison and indicates several important findings:

- The conventional (non-Dixon) LGE imaging technique is unable to discriminate fat from scar tissue.
- The infarct size calculated using mDixon images is similar to that estimated in routine LGE images.
- Lipomatous metaplasia is found only in myocardial segments with or directly adjacent to infarcted myocardium.
- Fat deposition does not influence regional myocardial function in segments with LGE, but has a positive effect on myocardial deformation in segments adjacent to scar tissue.

A number of case reports have been published that describe fat deposition in patients with chronic MI [19–21]. Many of these findings were unexpected, with unknown origin and clinical relevance. Fat deposition is common in the infarct area and is considered to be a part of the myocardial healing process [2]. Histological studies noted that fat tissue replacement can be detected in 78–84% of LV myocardial scars [22]. Cardiac CT and MR imaging studies also demonstrated high incidence of fat deposition, in agreement with autopsy findings [23, 24]. Moreover, it has been proven that noninvasive detection of lipomatous metaplasia could provide important prognostic information.

A number of CMR techniques may be used to detect lipid accumulation within the myocardium. A larger amount of fat tissue is visible in currently used bSSFP images as dark zones encompassing the borders between fat- and water-containing tissue due to chemical-shift artifacts [25]. We observed similar changes in MR signal intensity on cine images in 35% of patients included in our study. All these patients demonstrated fatty metaplasia on fat–water separated imaging. However, it remains unclear whether bSSFP images are robust enough to be used for detection of lipomatous metaplasia in routine practice. LGE imaging has been demonstrated to be a precise method to depict myocardial fibrosis [26]. However, fat is indistinguishable from fibrosis by conventional LGE imaging [27].

A method of fat and water separation based on proton chemical shift imaging was first described by Dixon [28]. Dixon showed that separate fat and water images can be generated by selecting two appropriate echo times for data acquisition where water and fat signal vectors develop progressive phases with respect to one another as a consequence of their chemical shift difference. The fat tissue is visible on the fat-only images, whereas water-only images enable the identification of edematous lesions or effusions [29]. Dixon’s original technique was hindered by several major limitations such as phase errors due to magnetic field inhomogeneity, long scan time and insufficient image quality [30]. Despite these challenges, substantial improvement and further technical developments enabled application of the mDixon technique in routine clinical practice. Recent advances ensure high spatial resolution images with good SNR, which can be acquired during short breath-hold scan.

**Fig. 6:**
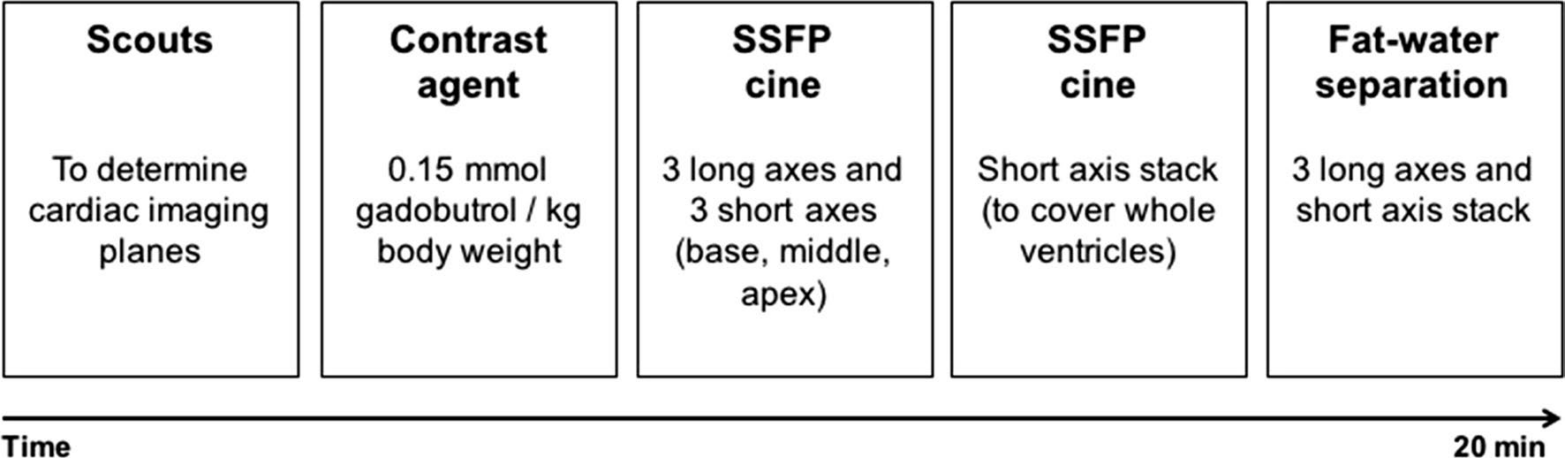
Practical schematic of proposed imaging protocol for patients with chronic MI to characterize myocardial tissue. Imaging protocol could be started with scouts to determine cardiac imaging planes following immediate gadolinium injection. After administration of contrast agent cine images could be acquired in three long-axis and stack of short-axis orientations. Functional imaging can be done in 10 min after which tissue characterization using fat–water separated imaging could be started. As 3D mDixon imaging ensures detailed tissue characterization this technique would be sufficient to complete myocardial viability protocol. *MI* myocardial infarction

We demonstrated that detection of fatty metaplasia only is possible with fat–water separated imaging. This has also been shown in previous studies. Furthermore, we showed that infarct size as determined with the mDixon technique is similar to the infarct size as estimated in routine LGE images. On the basis of our findings we propose an updated and shortened imaging protocol for patients with chronic MI (Fig. 6) using mDixon imaging. The important advantage of the current fat–water separated imaging technique is the ability to measure total fat volume in a 3D approach by segmentation of voxels that predominantly contain adipose cells [6]. Moreover, 3D acquisitions allow a reduction in imaging time as a single 3D long-axis stack scan requires only one breath-hold. This could help to improve diagnostic accuracy and add prognostic value while saving imaging and post-processing time.

The ability to quantify diffuse myocardial tissue alterations using noninvasive imaging is of considerable clinical interest. Parametric mapping techniques allow signal quantification by using T1 and T2 relaxation times that are displayed as color maps to facilitate visual assessment. The native T1 is increased with myocardial edema, fibrosis or deposition of amyloid and is reduced in lipid accumulation, Anderson–Fabry disease or iron overload [31]. Post-contrast T1-mapping reflects gadolinium concentration in the extravascular compartment and can be used to estimate extracellular volume fraction (ECV). However, a partial volume effect occurs within the myocardium in the case of fat accumulation; therefore, estimated values should be interpreted with caution [32].

Since fatty metaplasia was found only in segments within or directly adjacent to the infarct area, one might infer that the healing process is a precursor of lipid accumulation [24]. The studies reporting fat deposition location are conflicting. A recent CMR study showed that fat deposition was predominantly mid-myocardial or subepicardial, whereas cardiac CT studies report that adipose tissue almost always is detected in the subendocardial layer [23, 24]. We found that the majority of patients had transmural fat deposition.

CMR-FT as a novel tissue tracking technique became available in 2009 [18]. The advantages of CMR-FT are that the technique uses routine cine images and does not require acquisition of additional images therefore helps to save imaging time. The method had been validated against the traditional CMR tagging and speckle tracking echocardiography techniques [10, 33]. Comparative studies have reported that the most consistent parameters derived form CMR-FT are global circumferential and global longitudinal strain [18, 33].

It has been noted that myocardial segments with scar show lower deformation values than remote myocardium [18]. Notwithstanding, there are no studies assessing myocardial deformation parameters in segments with different tissue composition. We also showed that circumferential and radial strain values were significantly lower in segments with LGE extending to >50% of LV wall thickness. We did not observe significant differences in global circumferential and radial strain individuals with and without fat deposition. We assume that a much larger sample size is necessary to detect these subtle changes. It should be noted that fat tissue is detected in quite a low number of myocardial segments (in our study we found lipid accumulation in only 9.1% of all analyzed segments) and this may be an explanation why it did not influence global myocardial deformation significantly. In our study, we failed to demonstrate that fat deposition influences regional myocardial deformation in segments with LGE. However, we found that fat accumulation has a positive effect on myocardial function in segments adjacent to scar tissue.

Earlier studies indicated that myocardial fibrosis is the primary structural observation associated with ventricular tachycardia [34]. However, not all patients with MI experience ventricular tachycardia despite presence of intramyocardial collagen. The presence of fatty metaplasia may be a substrate for cardiac arrhythmias and sudden death; thus noninvasive detection of this structural remodeling could be of high prognostic value [35].

### Limitations

Several limitations of the current study should be mentioned. First, the population of this study was relatively small and there was a wide variety of MI characteristics in terms of age, treatment, and location. Subjects of the study were mostly long-term survivors with preserved LV systolic function. Second, the risk factors leading to fat deposition have not been investigated in this study.

## Conclusions

In this single center study we assessed characteristics of myocardial tissue in patients with chronic MI using mDixon CMR imaging and compared it with routine LGE technique. Fat–water separated imaging ensures more detailed tissue characterization in patients with chronic MI, without a relevant increase in imaging and post-processing time. Fatty metaplasia may influence regional myocardial deformation especially in myocardial segments adjacent to scar tissue. However, larger studies are necessary to prove this finding. These achievements may help to identify patients at risk for future cardiac events. A simplified and shortened myocardial viability CMR protocol might be useful to better characterize and stratify patients with chronic MI.

## Abbreviations

MI: Myocardial infarction
CMR: Cardiac magnetic resonance
FT: Feature tracking
LGE: Late gadolinium enhancement
ECV: Extracellular volume fraction
LV: Left ventricle/ventricular
LV EDV: Left ventricular end-diastolic volume
LV ESV: Left ventricular end-systolic volume
LV EF: Left ventricular ejection fraction
Ecc_LV_: Left ventricular circumferential strain
Err_LV_: Left ventricular radial strain
